# Gradient vortex dynamics in 3D-weak turbulence

**DOI:** 10.1038/s41598-025-94832-2

**Published:** 2025-09-30

**Authors:** Rubens A. Sautter, Reinaldo R. Rosa, Debora C. Alavarce, Kristian T. Spoerer, Flavio H. Fenton

**Affiliations:** 1 Academic Department of Informatics, UTFPR, Pato Branco, PR 85503-390 Brazil; 2 Lab for Computing and Applied Mathematics, COPDT-INPE-MCTI, S.J. dos Campos, SP 12245-010 Brazil; 3 HIPOCAMPUS , São José dos Campos, 12246-900 Brazil; 4 School of Computer Science, University of Nottingham, Nottingham, 12245-010 UK; 5 School of Physics, Georgia Institute of Technology, Atlanta, 12245-010 USA

**Keywords:** Physics, Mathematics and computing, Fluid dynamics

## Abstract

Vortex dynamics play a central role in most turbulent processes, whether of physical or chemical origin. In the realm of so-called weak turbulence, which encompasses physical, chemical, and electrochemical processes, understanding and monitoring the emergence of vortices in three dimensions remains a significant challenge. In this study, we propose a novel approach with minimal computational cost that enables the characterization of vortex ring formation and screw-like patterns in 3D-turbulent flows. Our method involves analyzing gradient vortex dynamics by measuring phase fluctuations in gradient patterns derived from the 3D-distribution of the corresponding amplitudes. The investigation focuses on transient primary structures generated by the Complex Ginzburg-Landau amplitude equation. The simulations integrate gradient pattern analysis, allowing for a groundbreaking association between phase fluctuations (commonly referred to as phase turbulence) and the helical oscillations induced by vorticity. As our main result, the phase-gradient analysis, combined with aspect ratio measurements of the primary patterns of coherent structures, enables us to identify at least four distinct regimes characterizing vortex dynamics. To further enhance this characterization, spectral measurements and recurrence plots of the phase-gradient fluctuations are introduced as innovative tools for describing weak turbulence and spatiotemporal chaos in nonlinear ($3D+1$) dynamics. This approach provides new insights into the intricate interplay between phase turbulence and vortex dynamics, offering a new perspective on the systematic study of the formation of coherent structures in three dimensions. It is worth highlighting that this is the first time that 3D screw dynamics have been simulated, visualized and analyzed in detail in a phase turbulence process.

**Supplementary Information:**

The online version contains supplementary material available at 10.1038/s41598-025-94832-2.

## Introduction

The formation and stability of vortices remain central challenges in fluid dynamics. Formal studies on the dynamics of such coherent structures in fluids began as early as the 1930s, primarily driven by the need to understand and describe the formation of cyclones and tornadoes across various meteorological scales [e.g.^1–6^]. However, significant progress was achieved in the 1970s^7,8^, when exact and approximate solutions to the axisymmetric Navier-Stokes equations for confined vortex flows were derived and validated through comparisons with experimental data. Over the following decades, these foundational studies led to the establishment of a robust theoretical framework for vortex dynamics in turbulent fluids, particularly in two-dimensional settings (see, e.g.,^9,10^ and references therein). Despite this progress, the study of three-dimensional vortex dynamics within the turbulence paradigm, based on the Navier-Stokes framework, has faced notable limitations. These challenges arise from the computational demands of rendering highly detailed numerical grids over extensive temporal and spatial domains, as well as the difficulty of establishing accurate initial conditions for vortex formation and the associated coherent structures^11^.

A significant breakthrough in this field was the incorporation of the concept of coherent structures into vortex dynamics in complex scalar fields [e.g.^12,13^]. This phenomenon, also observed in out of equilibrium dissipative systems, has been successfully modeled and simulated using reactive-diffusive approaches, such as the time-dependent Ginzburg-Landau model^14–18^, and the FitzHugh-Nagumo model among many other excitable systems. These advancements have provided new insights into vortex behavior, bridging gaps between fluid dynamics and the broader theory of dissipative systems.

In this work, we propose a new method to monitor and quantify the three-dimensional vortex dynamics based on Gradient Pattern Analysis (GPA)^19,20^. The performance of the method is evaluated through simulations of the complex Ginzburg-Landau equation^18,21–23^. The simulations are conducted using a minimal configuration, which enables the inspection of the entire dynamics of coherent structures across the spatiotemporal domain, characterizing regimes associated with chaotic dynamics^24^ and weak turbulence^25^.

### The amplitude equation

**Fig. 1:**
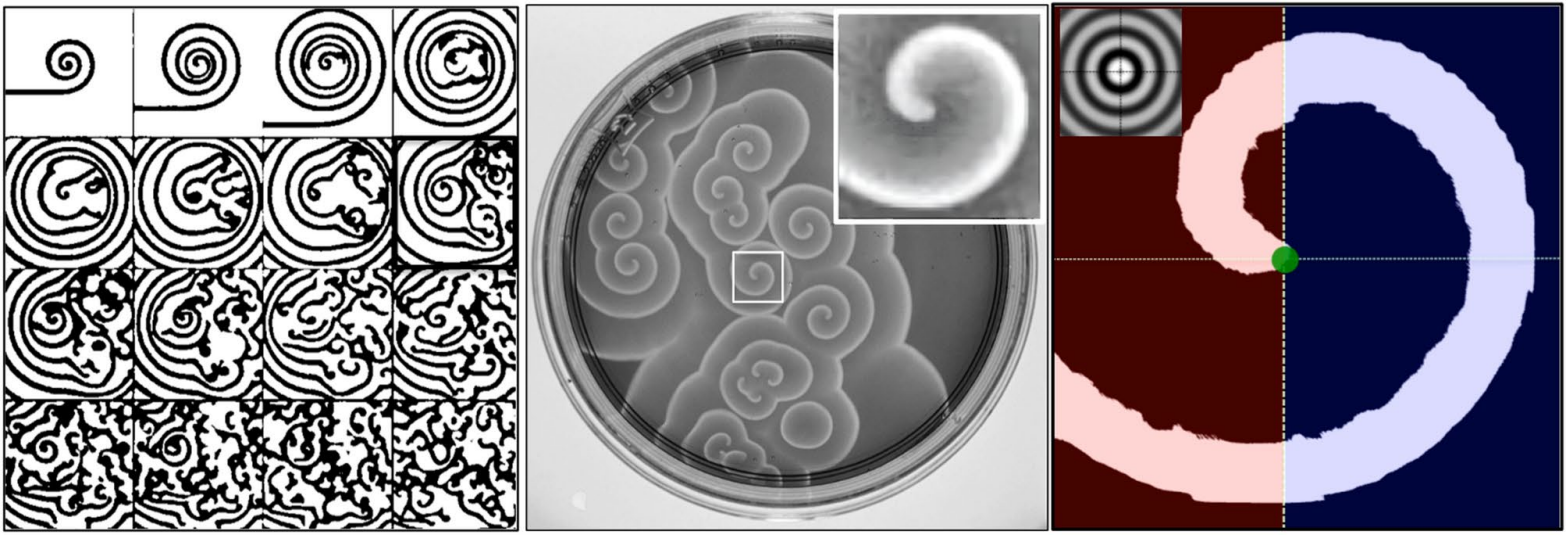
Characteristics of vorticity in Belousov-Zhabotinsky (BZ) reactive-diffusive dynamics. Left: sketch^28^ of the temporal evolution from an instability that generates a spiral flow in diffusion until reaching a disordered structure also called *phase turbulence*. Middle: Zooming one of the spirals in the BZ experiment (the BZ background image was provided by Dr. M. Hauser from Von Guericke University (Magdeburg, Germany)). Right: Binary image of the highlighted spiral identifying its property of bilateral asymmetry. In the inset at the top left, by comparison, the pattern known as *target* is shown, which is completely bilaterally symmetric (that is, in relation to the vertical and horizontal axes, taking the center of the pattern as the intersection).

Today, many fundamental principles of spatiotemporal pattern formation in dissipative systems are well understood theoretically and, in some cases, have also been demonstrated experimentally. However, significant challenges remain in understanding and tracking the dynamics of coherent structures within chaotic and turbulent flows. Particular interest lies in observing and describing the coherent structures that arise when active media are subjected to instabilities governed by nonlinear and stochastic dynamics^26^.These phenomena have been extensively studied in reactive-diffusive systems (RDS), where inhomogeneities manifest either as stable structures or as non-stationary, disordered patterns that can be characterized as turbulent^27^. A notable example is the use of amplitude equations^25,28–31^ to describe the emergence of Turing-like structures in the Belousov-Zhabotinsky (BZ) reaction^28,32,33^. This approach typically involves the complex $2D+1$ amplitude domain, where the temporal evolution of structures is studied in two spatial dimensions (2*D*) along with one additional domain (1) either amplitude or phase-selected for detailed pattern formation analysis. In these observed $2D+1$ dynamics, coherent states can persist in stable or metastable configurations^34–36^, giving rise to nonlinear concentration oscillations that may generate spiral waves (see Fig. 1). Such behavior is effectively captured by various models based on equivalent nonlinear amplitude equations^26^, among which the Complex Ginzburg-Landau Equation (CGLE) has proven particularly versatile in diverse applications^21–23,37^. The specific form of the CGLE relevant to our study can be expressed as follows:

1 $$\begin{aligned} \partial _t A = (1+ib) \nabla ^2 A + A - (1+ic) |A|^2A, \end{aligned}$$

where *A* is a matrix of complex numbers, *i* is the complex number $\sqrt{-1}$, *b* controls the spatial term and *c* is the reactive dispersion parameter.

The numerical solution of CGLE enables a precise formulation of spatiotemporal processes based on amplitude equations, which aids in identifying transitions between weak turbulence regimes and fully developed turbulence. Reaction-diffusion systems (RDS), which can also incorporate advection^38,39^, are crucial for understanding regimes such as spatiotemporal chaos^24^, a phenomenon that still lacks robust characterization. This challenge parallels the difficulty in fully describing turbulent flows in both neutral^40,41^ and ionized^42–44^ fluids in nature.

A key yet incompletely understood feature of spatiotemporal chaos in RDS, and Kolmogorov turbulence in fluids, is the emergence of transient vortex states^21,45,46^. These states, often associated with Turing-like patterns-such as spirals and vortex rings-appear at multiple scales and play a pivotal role in interpreting critical processes across disciplines. In physics, these patterns shed light on fluid and wave dynamics^27^; in chemistry, they are tied to reaction-diffusion phenomena, such as the BZ reaction^21^; and in biology, they hold significant importance, particularly in relation to heart dynamics^33,47,48^ where spiral waves and 3D scroll waves have been verified experimentally.

**Fig. 2:**
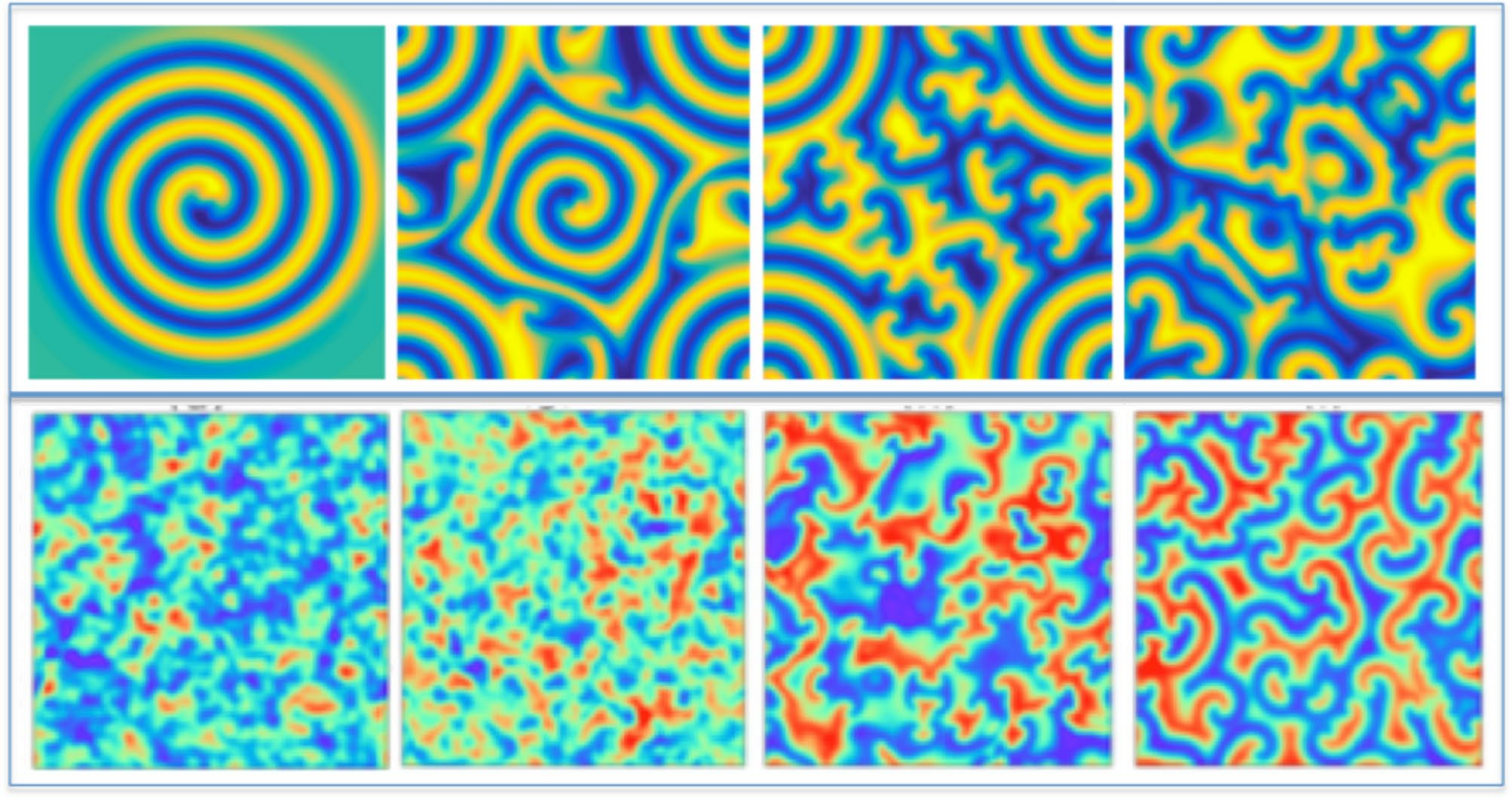
Examples of results obtained from simulations with ($2D+1$)-CGLE that result in 2D-weak turbulence (also known as defect turbulence) due to the reactive-diffusive process. Up: sequence of four snapshots taking a spiral as the initial condition. Bottom: sequence of four snapshots from a random initial condition. Clearly the top-down configuration (Up) maintains more bilateral symmetry than the bottom-up configuration (bottom). In the latter, due to spiral defects, bilateral asymmetry is more localized, increasing phase turbulence.

In the context of incompressible fluid dynamics, a recent breakthrough^49^ identified a vortex blob sustained by turbulent jets, exemplifying the existence of transient vortex states. This discovery mirrors the transient spiral state phenomenon observed in $2D+1$ CGLE simulations. Recently, as shown in Fig. 2, simulations developed from our CGL-algorithm in $2D+1$ corroborate these results^50^. Notably, this is illustrated in the second snapshot (from left to right) at the top of Fig. 2, highlighting the dynamic interplay of spiral formation and transient structures.

In addition to spirals, other coherent structures are also the result of reaction-diffusion waves that form between transients structures, when the system evolves dynamically over time, with the wavefront traveling and creating complex patterns, such as spirals, oscillations or chaotic structures. Due to the inherent nonlinearity of the reaction and diffusion terms, waves can exhibit a range of behaviors, from stable propagation to chaotic or turbulent dynamics. In addition to spirals, other examples of reaction-diffusion waves are: the propagation of flame fronts driven by chemical reactions^37^ and thermal diffusion^25^; reaction-diffusion waves that carry signals through cells^37^; propagation of electrical waves in the heart^47^, which can become irregular and cause arrhythmia; Patterns such as spots and stripes in the fur of animals can theoretically arise from reaction-diffusion systems^26^.

It is important to note that, due to computational and analytical limitations, most studies to date have focused on the $2D+1$ problem, with relatively few investigations addressing the more complex $3D+1$ problem. Therefore, the primary contribution of this research lies in the development of a comprehensive framework that integrates simulation and analysis in three dimensions, with a specific focus on detecting coherent structures emerging from reaction-diffusion waves that generate vortex patterns within amplitude hypercubes. The term *amplitude hypercube* ($3D+1$) refers to a discrete volumetric space where each coordinate in the 3D grid is associated with a numerical value representing the amplitude, *A*(*x*, *y*, *z*), at that specific spatial point. This concept extends the traditional notion of a 3D Euclidean grid by incorporating an additional dimension beyond the three spatial coordinates, enabling the representation and analysis of amplitude distributions across the entire volume in a higher-dimensional context.

### Computational challenges and contribution of this research

Vortex dynamics often involve highly nonlinear behaviors, making analytical solutions difficult. In turbulent-like flows, vortex interactions are chaotic and require advanced computational models to study. Most important, vortex structures span a wide range of scales, from microscopic to macroscopic, demanding multi-resolution techniques. Therefore, capturing vortex behavior experimentally or simulating it accurately, especially in 3D, is resource-intensive. In this framework, although there have been remarkable investigations^11,51–54^, both experimental and numerical, there is still a significant gap in approaches focused on the analysis of transient states in the $3D+1$ domain. Addressing this limitation requires progress along two complementary lines. The first pertains to the inherent challenges in conducting advanced real-world experiments to analyze vortex dynamics in 3*D* with precision and control. This issue, while critical, falls outside the scope of our present work. The second, and our primary focus, involves overcoming the limitations of effective numerical simulations in the $3D+1$ domain, particularly given the substantial computational complexity involved in inspecting each snapshot of a simulation in search of transients and regimes defined by metastability of coherent structures over time. The term *metastability* is used here to refer to the lifespan of a dominant structure that maintains spatial and temporal coherence throughout the weak turbulence process simulated using the CGLE.

In the computational context, implementing real-time measurement extraction presents significant challenges, largely due to the reliance on conventional metrics to characterize 3*D*-nonlinear spatiotemporal dynamics. From a general $3D+1$ perspective, where instabilities can drive the system out of equilibrium, this problem includes direct numerical simulation (DNS) of turbulence, chaotic systems, molecular dynamics in complex media, and N-body gravitational interactions. In all of these systems, a commonly used reference metric is the structure function, which typically relies on two-point correlation functions^55^. For discrete 3D volumes, the N-point correlation function encapsulates the spatial distribution of N-tuplets within a 3D lattice. However, its estimation scales as $n^{N}$, where *n* is the number of points. As *N* increases, this approach becomes computationally prohibitive. In addition, extracting precise metrics from a hypercube often depends on clustering algorithms, such as the friends-of-friends method, which is well-documented in N-body simulations in cosmology^56^. These methods impose additional computational costs, further complicating efforts to characterize 3D-vortex dynamics effectively.

Our primary challenge, therefore, is to develop a computationally efficient simulation strategy for 3D-weak turbulence, incorporating a measurement method capable of identifying and characterizing 3D-vortex dynamics without the high computational costs associated with traditional approaches. Therefore, the key contribution of this research is the development of a novel simulation framework that generates a gradient lattice, allowing for the efficient extraction of phase fluctuation metrics. This approach eliminates the dependence on structure-function methods and, through the incorporation of aspect ratio measurements, expedites the characterization of pattern formation regimes during the evolution of 3D-complex systems involving turbulence or spatiotemporal chaos.

### Organization of the paper

Building on the introductory content, this study presents a new simulation methodology for data generation along with an innovative analytical approach to explore the simulated data. The Research Methodology section provides a comprehensive overview of the research design and methods employed, complemented by a supplementary Methods subsection that details the specifics of each technique used. The findings are presented in the Results Analysis section, which outlines key insights derived from the study. A critical evaluation of the results, along with an in-depth analysis of the topic and its broader implications, is provided in the Discussion section. Finally, the Conclusion and Future Work section summarizes the research outcomes and offers recommendations for future studies. The simulation algorithm, the generated data (videos and hypercube files), and the codes for data analysis are available in the Additional Information section on Supplementary Material.

## Research methodology

The methodology comprises a robust CGLE simulation framework, selecting appropriate parameters, and running multiple tests to generate meaningful data. Data analysis involves processing these data using GPA to dynamically identify morphological patterns, validate the hypothesis of coherent structure formation, and derive insights. The approach is designed to provide a controlled and replicable way to study vortex dynamics from three-dimensional simulations.

### CGLE simulations

**Fig. 3:**
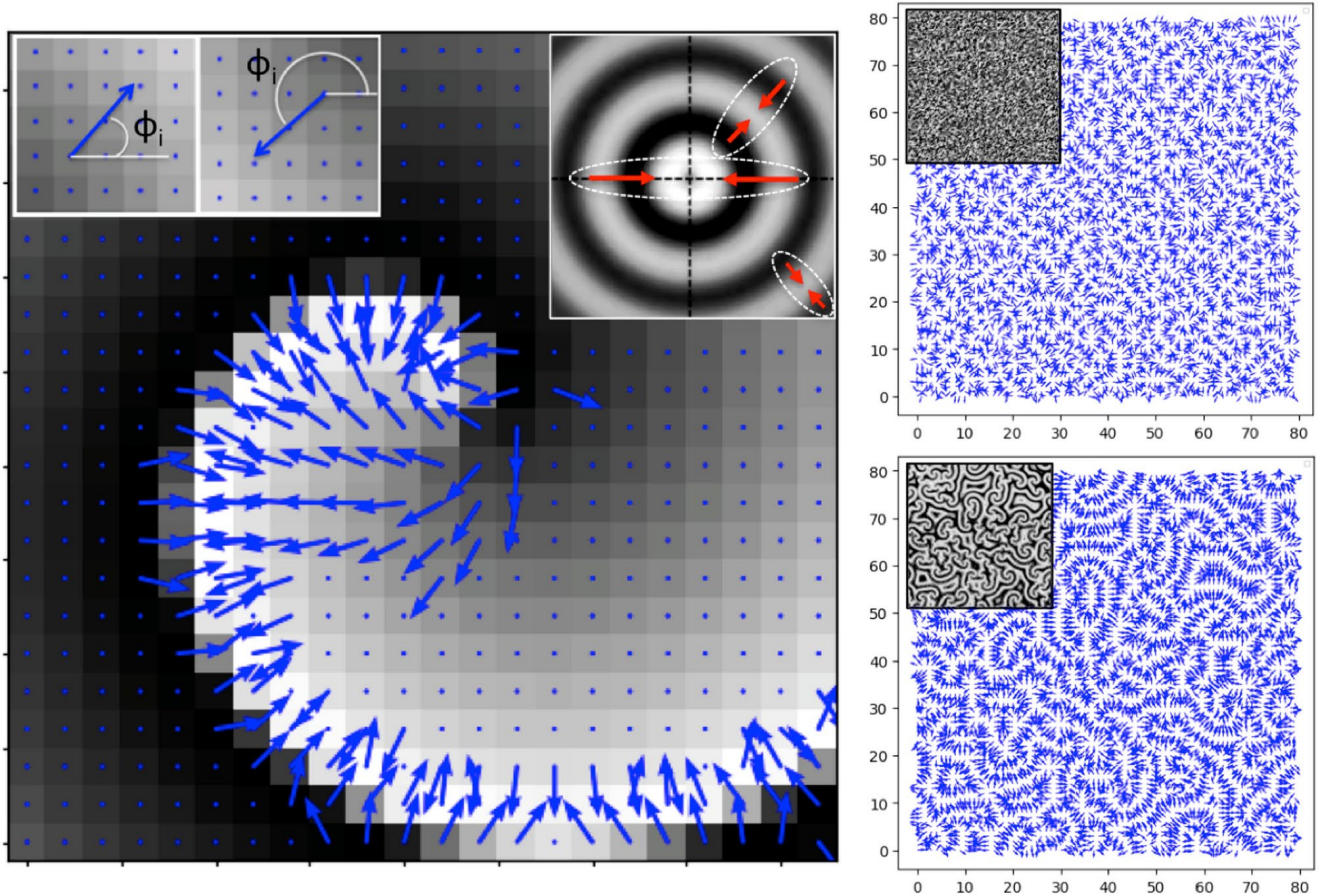
Pattern gradient lattices obtained with the GPA operation. Left: First helical structure from the tip of the spiral with the respective vector lattice superimposed on the image. In the inset at the top left, two generic vectors are shown with the criterion for measuring their respective phases from their horizontal baselines (applied to all asymmetric vectors (in blue)). In the inset on the right examples of symmetric vector pairs (in red) are shown for the *target* pattern which is completely symmetric (note that, for this particular symmetric pattern, $V_A$, as defined in equation 3, is null). Right: at the top, is shown the lattice gradient for a typical correspondent random pattern (in the inset picture). At the bottom, is shown a typical lattice gradient for phase turbulence (in the inset it is the correspondent turbulent pattern).

Using the Fourier Pseudo-Spectral Method (FPM)^57^, we successfully obtained high-quality solutions to the Complex Ginzburg-Landau Equation (CGLE), as illustrated in Fig. 2. Our earlier code for $2D+1$ simulations, which also incorporates noise terms, is detailed extensively in complementary work^50,58^. These simulations allowed for a comprehensive study of 2D vortex dynamics, where the formation of spiral defects is fundamentally governed by the bilateral asymmetry of the spiral pattern. This key property is effectively analyzed through the amplitude gradient, as shown in Fig. 3. Notably, our findings reveal that variations in the vector angles within the gradient lattice are closely linked to phase fluctuations of the reaction-diffusion waves. Consequently, alongside extending our simulations to the $3D+1$ domain, a critical challenge lies in advancing the phase analysis into the 3D-gradient domain. Our first significant challenge involved adapting the code for $3D+1$ simulations. This generalized code enables robust computation of spatial derivatives, allowing for detailed analysis of coherent structures, which are represented as an amplitude hypercube of appropriate dimensions (commonly 16$\times$16$\times$16 as a baseline^50^). Additionally, the FPM has recently demonstrated excellent spectral accuracy in solving the CGLE^59,60^, making it a highly practical and reliable method for this purpose.

In this FPM, the diffusion term is computed as follows:

2 $$\begin{aligned} \nabla ^2 u = \text {FFT}^{-1}(-4 \omega ^2 \pi ^2 {\widehat{f}}(\omega )), \end{aligned}$$

where $2\omega \pi$ represents the 3D spatial frequency, and ${\widehat{f}}(\omega )$ is the 3D Fourier transform (see more detail in methods section). The system parameters were selected to reproduce the main vortex dynamics found in weak turbulence highlighting, for future application purposes, the wave flows and its associated vortex dynamics. The relevant input parameters are summarized in Table 1. This table presents values derived from an extensive combinatorial search, accounting for all parameters. The values reported in this table allowed the simulation of the prototype in which the analytical gradient approach proposed in this work was carried out.

These values are those that resulted in the prototype simulation analyzed in this research.

**Table 1:** Parameter set for the CGLE solved in the $3D+1$ domain, where the integration tolerance $\tau$ comes from the method adopted to obtain numerical stability (see methods).

Parameter	Symbol	Value
Spatial dispersion parameter	*b*	0.00
Reaction dispersion parameter	*c*	1.50
Time-step	$\Delta t$	0.05
Spatial-step	$\Delta h$	1.00
Integration tolerance	$\tau$	$10^{-4}$
Grid-size	L	32×32×32
Initial condition	$A_0$	$\eta + i\eta$

These values are those that resulted in the prototype simulation analyzed in this research.

Our 3D simulator has, as its first output, the time-step sequence of *amplitude hypercubes*:

$$\begin{aligned} [A(x,y,z)_{1},\dots , A(x,y,z)_{i},\dots , A(x,y,z)_{M}]. \end{aligned}$$

**Fig. 4:**
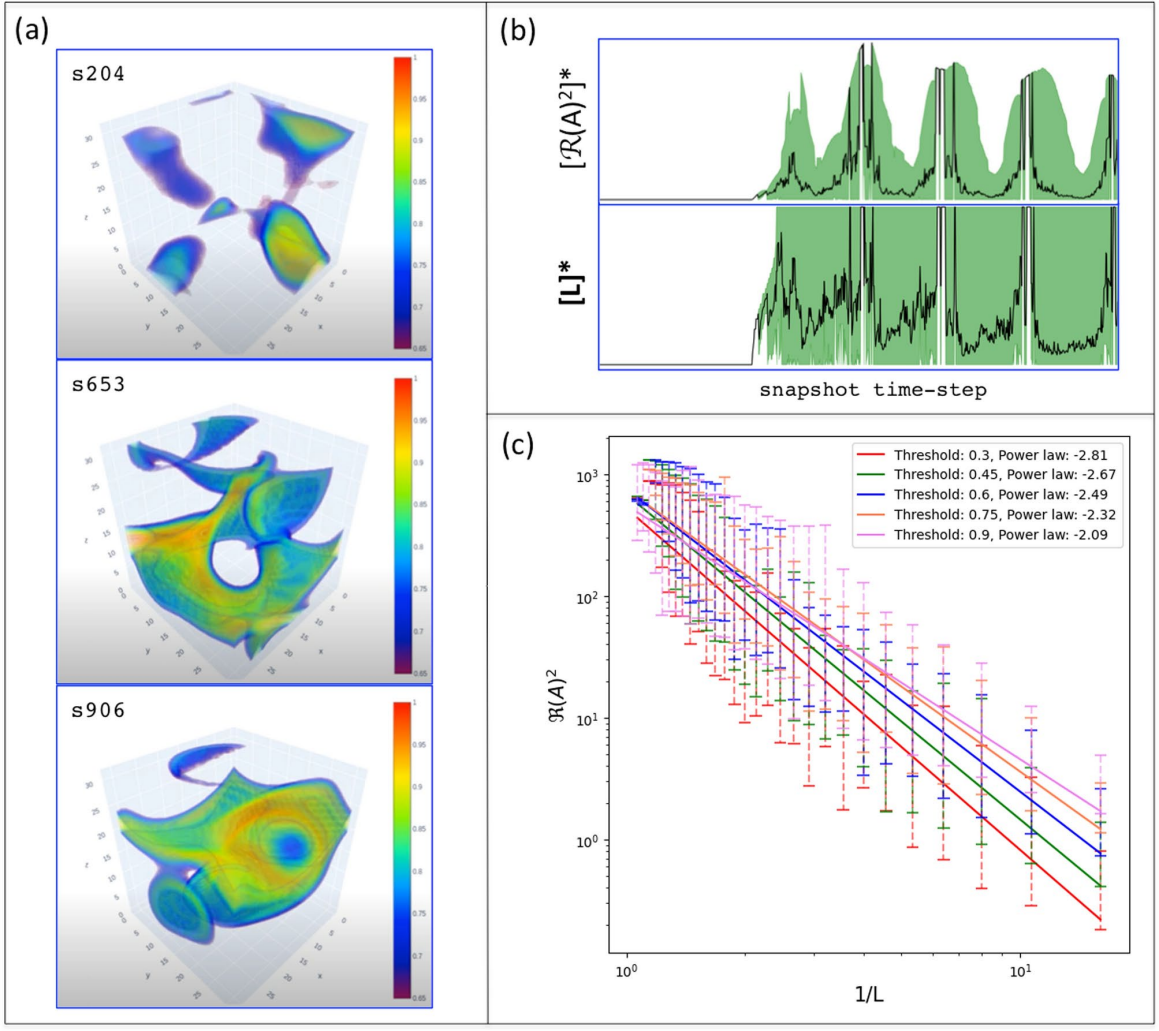
Validation of our ($3D+1$)-CGLE weak turbulence simulations. The complete time sequence is shown in *Movie Mv01* (available as supplementary material). **a**: Three representative snapshots of amplitude hypercubes showing typical coherent structures from vortex dynamics such as *irregular blobs* (s204), *whirls* (s653) and *rings*(s906); **b**: The corresponding time series for all snapshots of the simulation. The normalized mean amplitude (top) and the mean size of the corresponding structure (bottom), where the green area comprises the deviation from the mean size; **c**: The corresponding spectra representing the spatiotemporal amplitude fluctuation for weak turbulence in 3D.

Some representative snapshots from the simulation, performed for the purposes of this paper, are shown in Fig. 4a. The complete simulation can be viewed in supplementary material: Movie *Mv01*. The simulations were validated based on the pattern formation spectrum shown in Fig. 4c. We employed a two-step process involving thresholding and connected-component analysis^61^. In this methodology, we specifically targeted amplitudes exceeding a given threshold value. Subsequently, we applied a 3D-connected-component algorithm based on the 18-point, stencil approach^61^. To calculate the total structural energy, we sum the squared real parts of these selected amplitudes. This validation analysis allows us to obtain the average value (L) of the structures in each amplitude hypercube (Fig. 4a). The vortex dynamics of interest were observed from amplitude hypercubes with sizes $20\times 20\times 20$, reaching robust dynamics (rich in vorticity and metastable coherent structures) at size $32\times 32\times 32$. Therefore, for our purposes, the results shown in Fig. 4 validate our $3D+1$ simulations, operating as a low-cost computational prototype for this research.

**Fig. 5:**
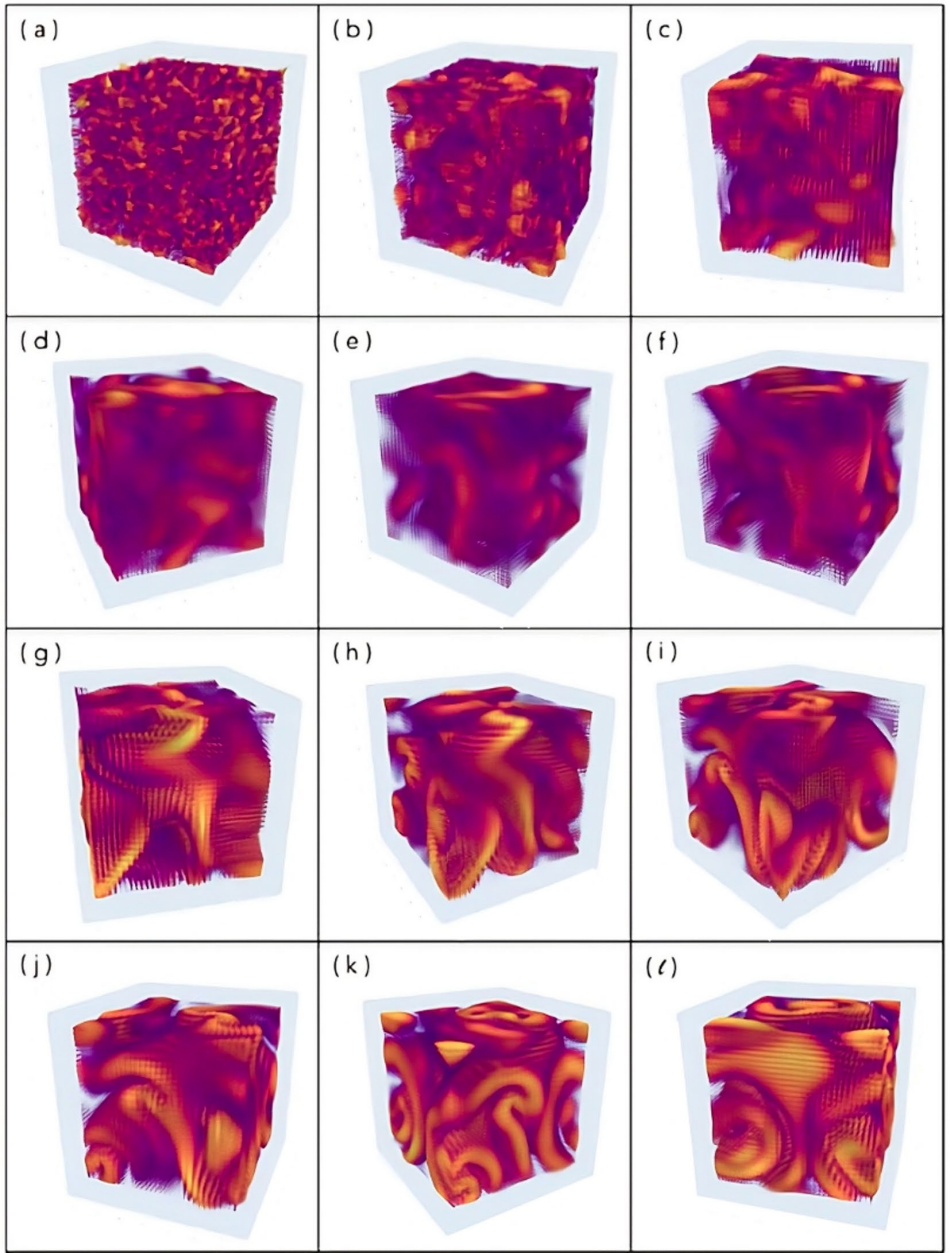
Gradient Hypercubes of ($3D+1$)-CGLE weak turbulence obtained from our simulations. The complete time sequence is shown in *Movie Mv02* (available as a supplementary material). Each gradient hypercube represents the output of the CGLE simulation. The simulation renders 1000 snapshots with a total duration of 30s in real time. The snapshots were selected considering the identification of four main regimes for the variation of ($\Gamma _{\ell }$): (I) initial regime with the formation of quasi-regular blobs with $\Gamma _{\ell }< 0.25$ for the range (0- 175) (**a**-**c**); (II) secondary regime characterized by inhomogeneous blobs with low vorticity with $0.25<\Gamma _{\ell }< 0.33$ for the range (176-325) (**d**-**f**); (III) high vorticity regime characterized mainly by screws and vortex rings with $0.33<\Gamma _{\ell }< 0.75$ for the range (326-575) (**g**-**i**); (IV) regime with vortex rings and inhomogeneous blobs with $\Gamma _{\ell }$ close to one for the final range (576-1000) (**j**-**l**). To monitor all the dynamics and inspect morphological details in particular snapshots, we recommend interacting with the data in video format (see supplementary material).

Alternatively, our 3D simulator has also as a complementary output the time-step sequence of *gradient hypercubes*:

$$\begin{aligned} [\nabla A(x,y,z)_{1},\dots , \nabla A(x,y,z)_{i},\dots , \nabla A(x,y,z)_{M}], \end{aligned}$$

which allows the unprecedented approach with gradient phase analysis that we propose in this report (see Fig. 5).

Regarding the gradient hypercube simulations, for each $A(x,y,z)_{i}$, the vector at each grid point was obtained from the real part of the neighboring amplitudes ($\Re [A]$). The construction of the 3D-gradient lattice, $\nabla A(x,y,z)$, is carried out adopting the same criteria as the GPA 2D-method (which takes the central derivatives (dx, dy))^58,62^, but addressing now (dx,dy,dz) to perform the 3D-gradient (see more details in methods section).

### Gradient phase analysis

The phase-gradient formula is derived by extending the original formula (from Gradient Pattern Analysis - see references in the paper), a widely used one, from a 2D vector field to a 3D configuration.

In the 3D case, an additional angle is introduced due to the spherical geometry adopted in the formulation. This configuration, tested and validated using canonical patterns (such as those illustrated in Fig. 9a-c), enables the quantification of bilateral symmetry breaking, analogous to the measurements performed in 2D.

The gradient phase analysis method presented here has been generalized from Gradient Pattern Analysis (GPA), which is a mathematical-computational formalism proposed to characterize two-dimensional spatial patterns based on the gradient lattice of a given amplitude matrix^19,20,63^. The gradient lattice of the matrix allows the extraction of metrics called *gradient moments*^58,62^ (see methods). All metrics are generally calculated on asymmetric vectors after removing all symmetric pairs, which are those that have the same values for the modules and opposite values for the phases. The third gradient moment (called $G_{3}$) is defined as being $G_{3}={V_A\over V} \left( 1 - \sum \frac{\phi _i^A}{2\pi }\right)$, where $\phi _i^A$ represents the phase of each asymmetric vector in the gradient lattice, *V* is the total number of vectors, $V_A$ is the number of asymmetrical vectors, and $v_i^A$ is the set of asymmetrical vectors. Therefore, if all vectors are symmetrical, $G_{3} = 0$. When all asymmetrical vectors are aligned, $G_{3} = V_A/2V$, while in a scenario, where vectors are in all directions, $G_{3} = V_A/V$. Therefore, the latter term is a measure of vector lattice alignment. The ability of $G_{3}$ to characterize spiral patterns has been established in recent applications for galaxy morphology^62^ and also to 2D-CGLE^58^ providing an effective new measure to characterize the spatial oscillatory dynamics from 2D-weak turbulence.

For the gradient phase analysis in 3D we adapted the $G_{3}$ measurement, which we simply call $G_{\phi }$ here, extending the same methodology used in 2D to 3D (see Fig. 10), where the spherical coordinate system is considered (see the methods description to find the technical details of this approach). The equivalent gradient phase in 3D is given by the following equation:

3 $$\begin{aligned} G_{\phi } = \frac{V_A}{V}\left( 1 - \sum ^{V_A}_{i=1} \left( \frac{\phi _{xy}^i}{2\pi } + \frac{\phi _{xz}^i}{2\pi }\right) \right) \end{aligned}$$

where the 3D spherical coordinate system imposes now the use of two complementary angles ($\phi _{xy}, \phi _{xz}$). Note that, *xy* defines the plane from which one of the angles is measured, the same applies to *xz*. Note that this criterion is equivalent to that adopted to obtain the phase of a vector in the 2D gradient lattice, as shown in Fig. 3 (inset at top left). The algorithm for calculating $G_{\phi }$ is available as a new operation module of the GPA code (see Additional Information). The $G_{\phi }$ value was obtained for all gradient hypercubes generated in the simulation described in the previous section, where some snapshots are shown in Fig. 5.

### Complementary analysis

In addition to the GPA-3D presented here, our approach incorporates well-established traditional analysis techniques succinctly described in this section as complementary methodologies.

#### Aspect ratio

Aspect ratio is a relative scaling measure that allows us to extract information about the shape of a structure, which can be critical for object classification, anomaly detection, and pattern recognition. To help interpret gradient phase fluctuations $G_{\phi }$ over time, we used simultaneous measurements of the aspect ratio $\Gamma _{\ell }= \ell /L$ (the ratio of the largest linear size ($\ell$) of the substructure to the size of the side of the volume, *L*), as it has been defined in applications for the analysis of structures in porous media^64^. Information about the technique and algorithm applied in this research is detailed in the Methods section.

#### Time-delay embedding

The technique of time-delay embedding (TDE), sometimes referred to as *recurrence plot*, was considered in order to reconstruct the simplest higher-dimensional representation of the $G_{\phi }$ time series to uncover its dynamics. This is a fundamental approach in nonlinear time series analysis and is used to visualize underlying structures, periodicity, or chaotic behavior in the data. To obtain the TDE portrait, the time series $G_{\phi }$ is plotted on the x-axis, and itself, shifted by a chosen delay $\tau$, is plotted on the y-axis. The portrait consists of points in the plane $(G_{\phi }(t),G_{\phi }(t+\tau ))$ . In our application, we consider identifying the different regimes by coloring the points as different patterns prevail over time from $G_{\phi }$ and $\Gamma _{\ell }$ inspection.

#### Spectral analysis

The $G_{\phi }$ time series was also analyzed using Power Spectral Density (PSD) and Detrended Fluctuation Analysis (DFA) in a composite approach^65^. The frequency content of the signal was first verified using a Fast Fourier Transform (FFT), confirming the primary frequency components of the data. The PSD method characterizes the distribution of power across frequencies, allowing for the estimation of spectral properties and the identification of the correspondent power-law behavior. DFA is employed to examine long-range temporal correlations within the signal, quantifying scaling behavior and identifying self-affinity properties. Together, these methods provided complementary insights: PSD highlighted the frequency-dependent dynamics, while DFA captured time-domain correlations and trends.

## Results analysis

**Fig. 6:**
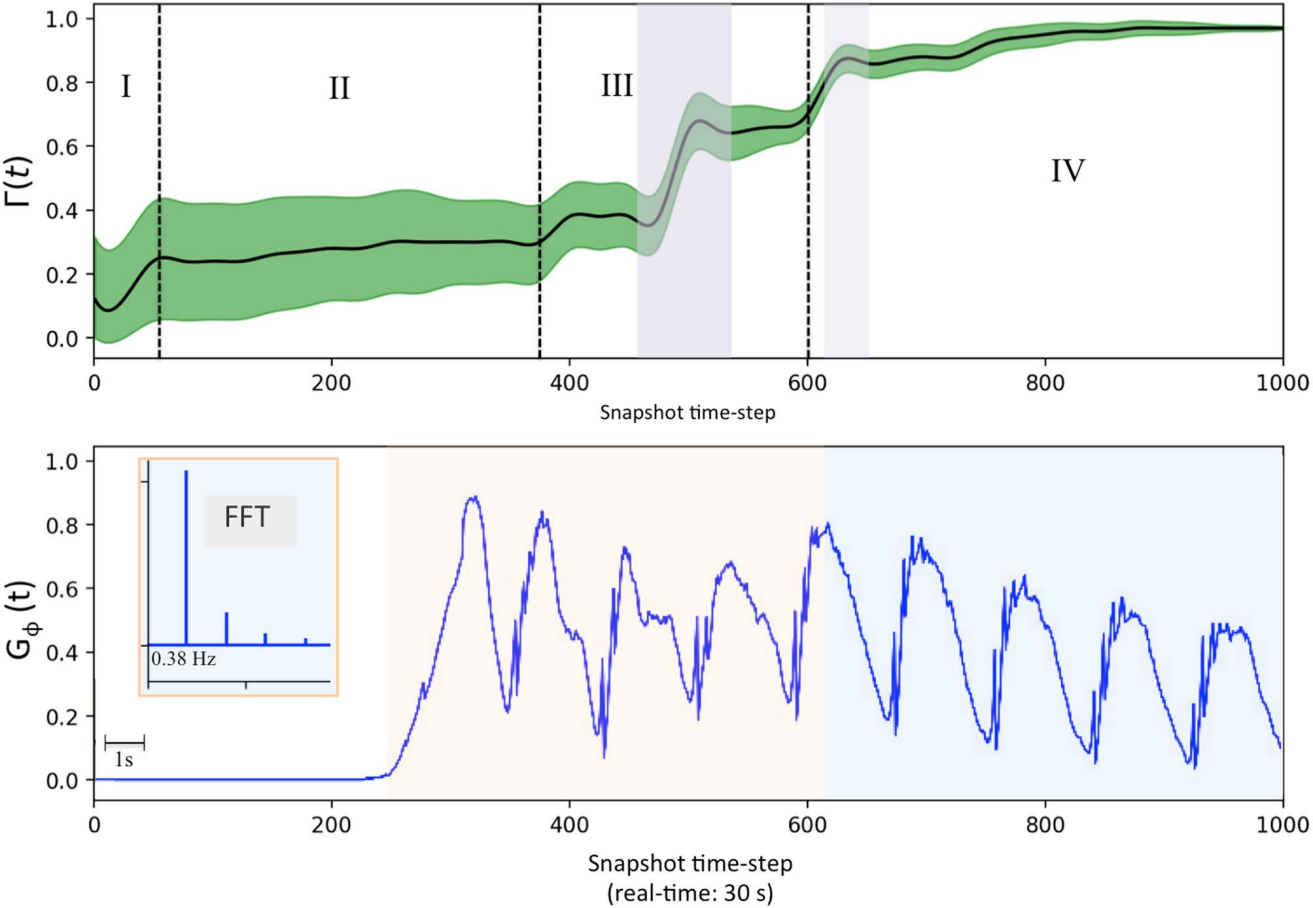
Outputs from the analysis of weak turbulence dynamics simulated with ($3D+1$)-CGLE. Up: Variation of the aspect ratio, $\Gamma _{\ell }$, over time. Average values are presented for each set of 25 snapshots making up 40 representative measurements. The bias range is represented by the variation in standard deviation over time. Four main regimes are identified and separated by black dotted lines. The identification of the four regimes, in a $32\times 32\times 32$ simulation, was the main result that validated this simulation for the analysis of the phase-gradient. The translucent violet bands identify the occurrence of the main screws (the most striking of them with $\Gamma _{\ell } \approx 0.65$ (s465) shown in detail in Fig. 8). Bottom: Values of $G_{\phi }$ calculated for each gradient hypercube. The result shows that the typical oscillations of vortex dynamics begin during regime II. Until approximately the end of regime III there is greater instability (orange translucent band) until the beginning of regime IV which imposes a decrease in the peak of the oscillations resulting in greater stability of the phases without the presence of asymmetries imposed by thinner vortexes. The values of $G_{\phi }$ fluctuate as more pairs of symmetric vectors are disregarded from the calculation of $G_{\phi }$. Table 2 presents representative average values for the main emerging patterns identified in this study. In the inset, the average frequency of the vortex dynamics represented by the fluctuation of $G_{\phi }$ was obtained with the Fast Fourier Transform (FFT), resulting in 0.38*Hz* for 30 seconds of observation taking only the oscillation band (colored part of the time series that starts at time-step=247).

A representative output of ($3D+1$)-CGLE weak turbulence is presented in Fig. 5. The simulation generates 1000 snapshots over a total duration of 30 seconds in real time. The full time sequence is provided as supplementary material in *Movie Mv02*. For each time-step snapshot, the phase gradient $G_{\phi }$, as defined by Eq. (3), is computed using the phase values from the entire dataset. Additionally, the aspect ratio $\Gamma _{\ell }$ is calculated based on the gradient hypercube, as detailed in the Methods section. The resulting time series for both measurements are displayed in Fig. 6. Notably, the dynamics observed in the gradient hypercube (Fig. 6, Movie 02) provide enhanced identification of screw structures and vortex rings compared to those in the amplitude hypercube (Fig. 5, Movie 01). In particular, from the gradient visualization, the formation of screw structures can be closely tracked during the snapshots intervals: from s370 to s400 and from s440 to s536.

### Gradient fluctuations analysis and structure identification

The joint analysis of the times series $\Gamma _{\ell }(t)$ and $G_{\phi }$, enables the identification of key coherent structures and their metastability during the weak turbulence pattern formation. By examining the temporal variation of the aspect ratio $\Gamma _{\ell }$ and quantitatively characterizing each type of structure, it becomes possible to infer the regimes corresponding to different transient phases. Furthermore, the identified regimes provide an initial framework for interpreting the finer structures observed within the phase-gradient time series $G_{\phi }$. As a result of the $\Gamma _{\ell }(t)$ behavior and the counting of coherent structures from the gradient hypercube, the following regimes were identified:

(I) An initial regime characterized by the formation of quasi-regular blobs, with $\Gamma _{\ell }< 0.25$, in the range he range (0-175) (a, b, c);
(II) A secondary regime featuring inhomogeneous blobs with low vorticity, where $0.25<\Gamma _{\ell }< 0.33$, in the range (176-325) (d,e,f);
(III) A high-vorticity regime dominated by screws and vortex rings, with $0.33<\Gamma _{\ell }< 0.75$ , in the range (326-575) (g,h,i);
(IV) A final regime consisting of vortex rings and inhomogeneous blobs, with $\Gamma _{\ell }$ approaching one, in the range (576-1000) (j,k,l).

As illustrated in Fig. 6, the values of $\Gamma _{\ell }$ fluctuate as the number of symmetric vector pairs is increased from the gradient lattice. The average frequency of the vortex dynamics, represented by the time series of gradient phase fluctuations, was calculated using the Fast Fourier Transform (FFT). This analysis revealed a dominant frequency of 0.38 Hz within the oscillation band. The quantitative characterization of these regimes is summarized in Table 2. The fourth column of Table 2 displays the volume proportions of each structure. These proportions were calculated using the ENVIRaster tool^66^, with further details provided in the complementary Methods section.

**Table 2:** Typical dynamical regimes found, their respective aspect ratio, gradient phase characterization, and the respective percentage of each type of structure in the Volume.

Regime	$\Delta \Gamma$	$\Delta G_{\phi }$	% in the Volume
Type I	$< 0.25$	0	Regular blobs $\approx 82\%$
Type II	$0.25-0.33$	$0 - 0.85$	Inhomogeneous blobs $\approx 76\%$
Type III	$0.33-0.75$	$0.10-0.80$	Screws $\approx 42\%$, Vortex Rings $\approx 54\%$
Type IV	$0.75-0.97$	$0.12-0.76$	Vortex Rings $\approx 38\%$, Inhomogeneous blobs $\approx 54\%$

**Fig. 7:**
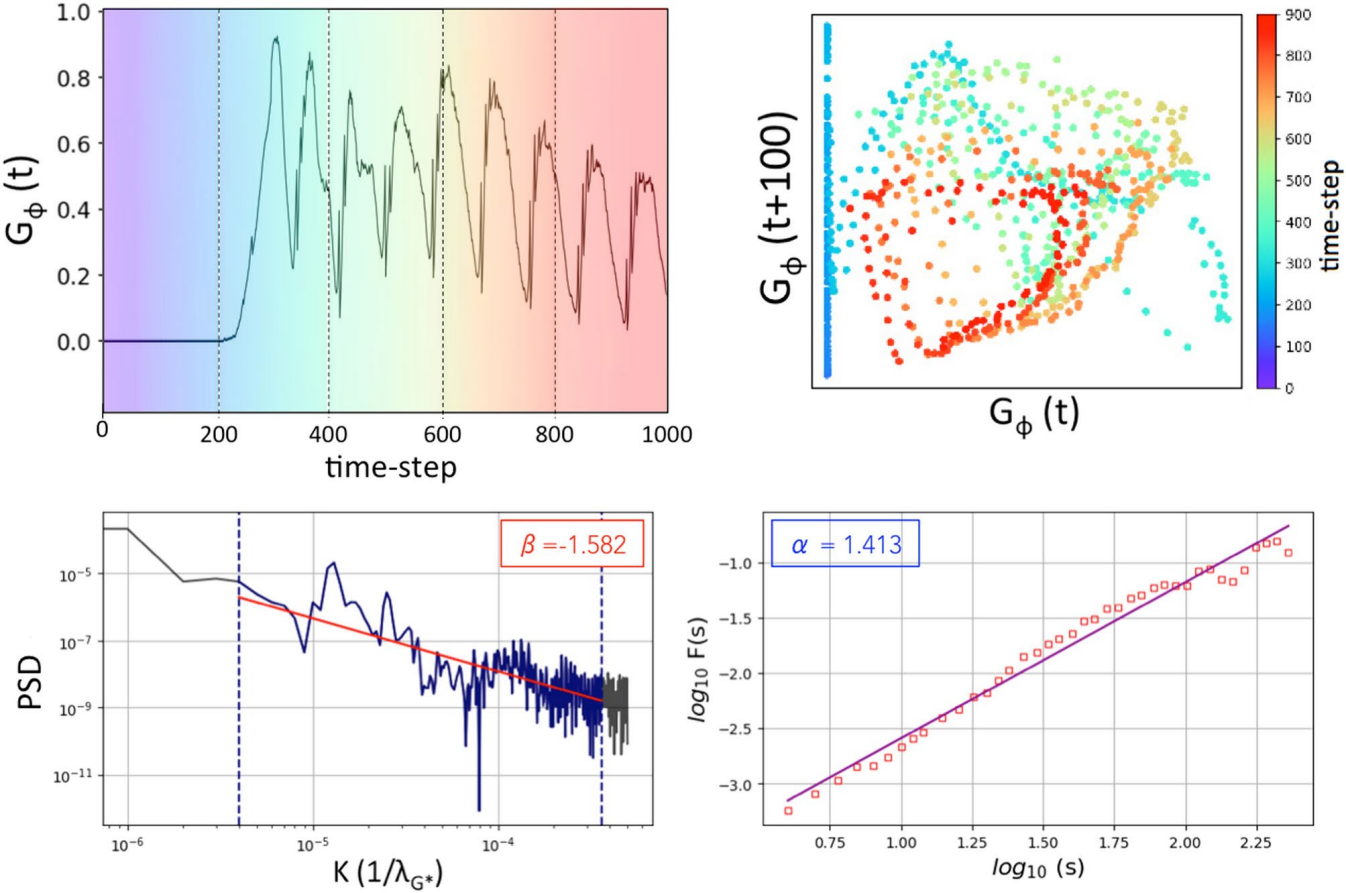
Characterization of the vortex dynamics process based on phase-gradient fluctuation. Up-Left: Time series of $G_{\phi }$ nuanced correspondingly to its time-delay embedding (TDE) shown in the figure alongside. Up-Right: TDE portrait with delay equal to 100. Value chosen by the best time interval criterion comprising at least three wavelengths ($\approx 1/120 Hz$) to configure a state space where a possible chaotic attractor may exist. Bottom: Spectral analysis of $G_{\phi }$ based on the Wiener-Khinchin approach. The richness of scales in the fluctuations is characterized based on the spectral indices, $\beta$ and $\alpha$, obtained respectively in two complementary spectral spaces, the Power Spectral Density (PSD) and the Fractal Spectrum via Detrended Fluctuation Analysis (DFA). The three outputs (from Recurrence Plot, PSD and DFA) serve as a first proposal set of measures to analyze the 3D vortex dynamics using the method presented in this paper.

As shown in Fig. 7, the time delay embedding (TDE) for $G_{\phi }(t)$ was constructed. The delay ($\tau =100$) was selected using the optimal time step criterion, ensuring that it encompasses at least three characteristic wavelengths to configure an appropriate state space. This result suggest a possible signature of a chaotic attractor based on the *gradient phase dynamics*. In the context of Dissipative Systems Theory, we analyze the shared properties of equivalent concepts such as *phase turbulence*, weak turbulence, and spatiotemporal chaos. These phenomena are characterized by irregular and complex behaviors spanning both spatial and temporal dimensions, driven by weakly nonlinear interactions. Specifically, the nonlinear coupling between *phase* and amplitude is a key mechanism underlying the emergence of irregular patterns in *phase dynamics*^25^.

Considering a Wiener-Khinchin approach previously adapted to our analytical context^67^, a complementary spectral analysis was performed on $G_{\phi }(t)$. The results are shown in Fig. 7 (Bottom). The fluctuations scaling was characterized through the spectral indices ($\beta \approx -1.6$ and $\alpha \approx 1.4$), obtained respectively from the Power Spectral Density (PSD) and the Fractal Spectrum via Detrended Fluctuation Analysis (DFA). Notably, the coherence between the spectral values derived from the amplitude and gradient hypercubes highlights the reliability of the spectral indexes, $\beta$ and $\alpha$, in this complementary analysis. Therefore, the results shown in Fig. 7 suggest that these three outputs from $G_{\phi }$, when combined, represent a promising first step toward the quantitative characterization of *phase turbulence* from gradient vortex dynamics, reinforcing their potential effectiveness for further applications.

### Screw analysis

#### The unprecedented capture of screw pattern formation

**Fig. 8:**
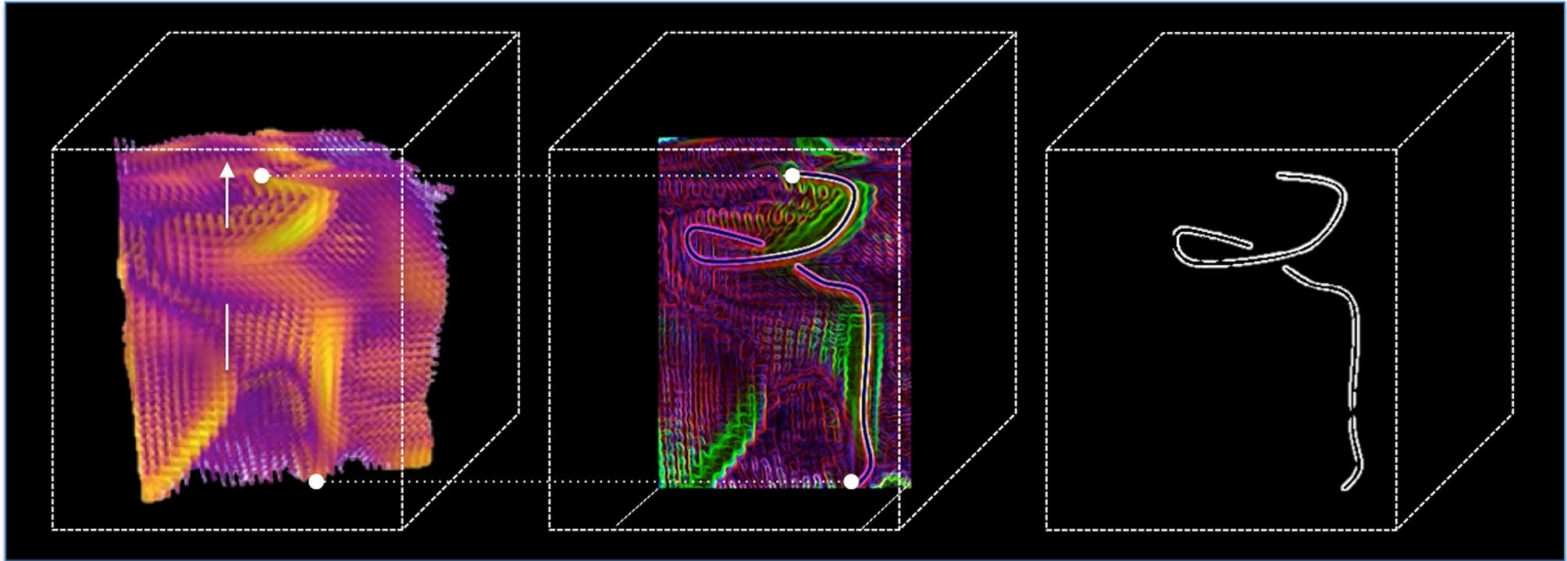
Detection and segmentation of the screw pattern in Snapshot 465. Identifying a screw pattern in the simulation: Left: Given a snapshot, its 2D projection is taken and input into a filtering cube based on the “dist” function from the Scipy.spatial library (python). From two points and an arrow determined by the user, the Yolo 7 smart filter (trained to recognize a screw) is applied. Middle: Identified structure is highlighted by filtering. Right: The background is removed and a binary filter is applied completing the identification. This procedure allowed us to analyze a possible cause of the increase in phase asymmetry, due to the oscillation of the screw axis discussed in Fig. 9.

As a key result, we present a graphical analysis of the screw pattern identified for the first time in this type of simulation. As illustrated in Fig. 8, the screw’s shape is effectively highlighted using computer vision techniques. This approach offers a compelling perspective that bridges the fields of computer vision and computer graphics. By leveraging a series of pre-analyzed gradient hypercubes, it becomes possible to employ computer graphics techniques for an immersive fly-through exploration of the gradient structure, enabling the identification of local asymmetry values associated with the visualized topologies. This methodology represents a significant analytical advancement for the study of complex systems in three dimensions.

#### Screw conjecture

**Fig. 9:**
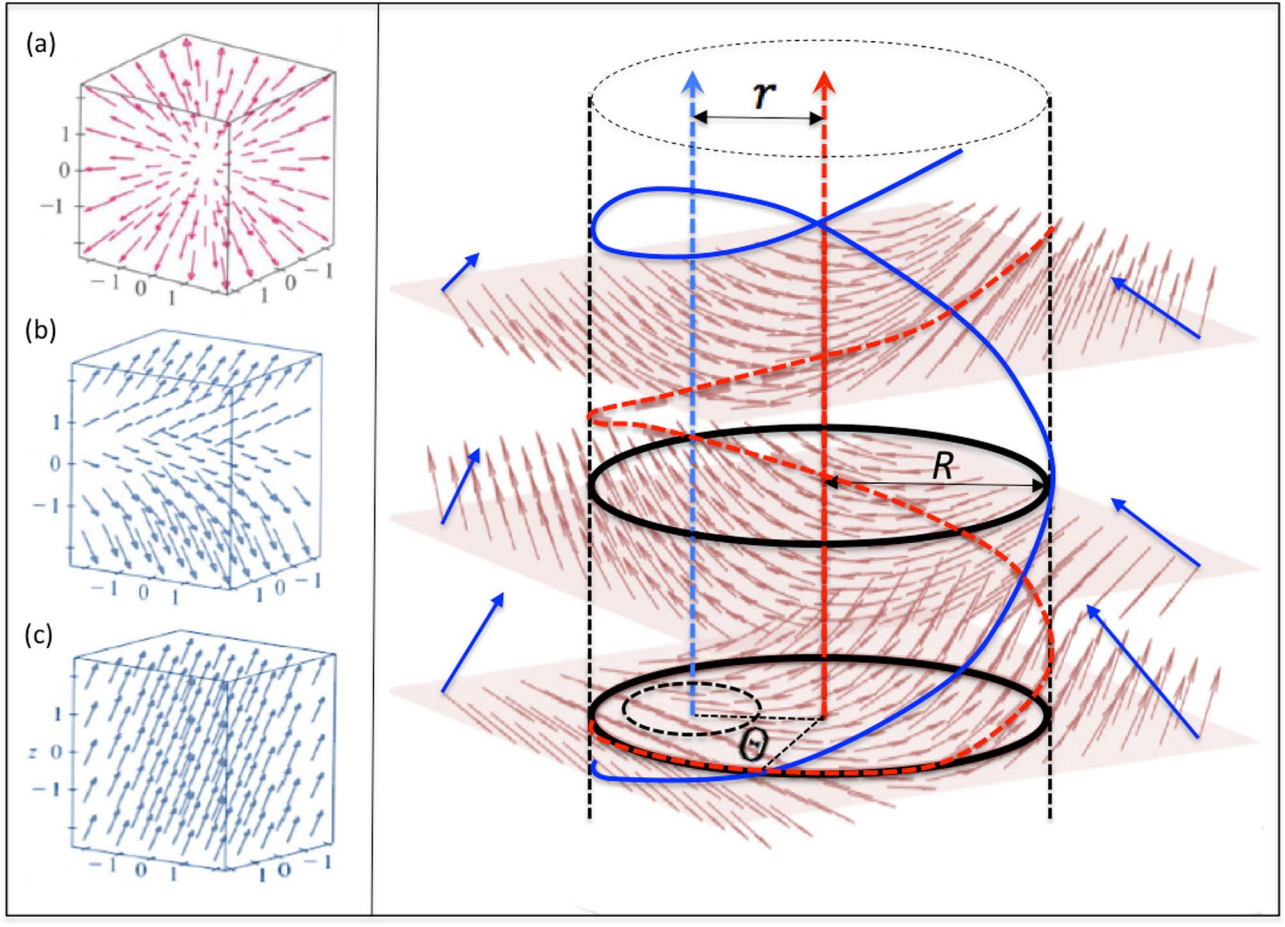
Influence of asymmetric screw vortex on the configuration of the gradient lattice. Left: Examples of basic gradient flows that are fully symmetric (**a**), and bilaterally asymmetric with a central sheet (**b**), and fully laminar (**c**). Right: a screw with a symmetric axis (sub-layers in red) presents smaller bilateral asymmetry than the blue screw pattern identified in the simulation (Fig. 8). The distance (r) and the rotation angle ($\Theta$) from the blue axis can be conjectured here as one of the causes driving the values of the phase-gradient ($G_{\phi }$) determining the complex oscillation regimes found in our results (Fig. 6).

In the time domain, as depicted in Fig. 9, geometric measures can be used to characterize the causes of the observed oscillations. Specifically, in the case of screw dynamics, we hypothesize that the oscillations in $G_{\phi }$ arise from variations in bilateral asymmetry due to shifts in the screw axis. This hypothesis positions the approach presented here as a promising method with potential for further exploration across diverse application contexts.

For instance, we propose analyzing the symmetry of screw patterns by tracking the temporal evolution of the parameters *r* and $\theta$ (Fig. 9, right). Achieving this, however, requires a Lagrangian tracer to capture the variation of these parameters. Such a strategy, to be considered in a complementary work, would enable an investigation into how asymmetric screw patterns directly contribute to the increase and oscillation of $G_{\phi }$, ultimately addressing the conjecture proposed in this study.

## Discussion

Characterizing the process of pattern formation in fluid systems is essential for understanding a wide range of complex phenomena governed by reaction-diffusion mechanisms, spatiotemporal chaos, and turbulence. Visualizing and analyzing three-dimensional mixing patterns are particularly critical for uncovering the nonlinear phenomena often observed in nature. However, progress in this area remains limited. To address this, the present study focuses on applying Gradient Vortex Dynamics (GVD) to weak CGL turbulence, as proposed herein. Specifically, it investigates the formation and interaction of coherent structures within three-dimensional wave flows driven by reaction-diffusion dynamics. These coherent structures play a pivotal role in determining the geometrical transport properties of the flow, making their formation and topological characteristics fundamental to understanding 3D-mixing phenomena along the whole process.

This research, finding a computationally optimized CGL model with GPA integration, significantly enhanced the visual monitoring of 3D dynamics throughout the process. According to the relevant literature, this approach enabled the unprecedented detection of screw pattern formation within a three-dimensional phase turbulence process. This finding represents a major advancement and highlights the potential for applying GVD to similar purposes in other fields or applications.

**GVD from more complex systems:** Through numerical simulations enhanced by three-dimensional gradient analysis and supported by analytical methods-including phase fluctuation metrics and aspect ratio measurements-this study enables precise monitoring of coherent structure formation. The results demonstrate the universal applicability of the proposed methodology and highlight its effectiveness in advancing the understanding of three-dimensional nonlinear processes. Additionally, this research offers a foundation for the experimental validation of candidate models and opens avenues for extending the method to other fluid dynamics applications. These include five-dimensional systems^68^ and cases involving more complex initial conditions, such as the Taylor-Green Vortex^36,69,70^.

**Cardiac Dynamics and Deep Learning:** The study of cardiac dynamics also has great potential for applications. Since there are models for blood pressure measured in different physiological conditions^71^, such models can be improved by describing cardiac behavior based on the 3D-dynamics of the left ventricle. As is well known, this study is directly associated with the vortex dynamics identified here by the phase-gradient method^33,47,48^. As coronary visualization techniques become more sophisticated, analytical methods such as the one presented here will act as a tool for monitoring and identifying 3D patterns not only in medical physiology but also in any other area that requires 3D visualization of complex dynamics. As a final comment, it is important to clarify that measures to characterize spatiotemporal nonlinear processes are limited. Even more scarce are those that can act as possible attributes and labels within the scope of supervised deep learning models^72,73^. In this case, intelligent monitoring via a DeepGVD is an incipient, necessary, and disruptive applied research topic, as is the monitoring of cardiac dynamics in 3D.

**Main Limitation of the Current Approach:** In weak turbulence, the study of the spectral index in energy cascades allows us to identify regions of the spectrum where there is dissipation (smaller scales) or accumulation of energy (larger scales) in coherent structures such as vortex rings, screws, and blobs that arise from the spatiotemporal dynamics. In this case, different spectral indices can distinguish between processes such as passive diffusion (dominated by advective transport) and more complex dynamics with strong nonlinear interactions resulting from reaction-diffusion that give rise to spirals and screws. However, the interpretation of dynamics from energy cascades in weak turbulence is still a subject of debate (especially in the spatiotemporal domain) to determine which spectral values indicate whether a direct cascade from large to small scales occurs. In this context, our approach, while still in its early stages, shows significant potential to establish new criteria based on the phase-gradient spectral index. To advance this work, it is essential to enhance the interpretation of the spectral indices obtained and validate their behavior using additional simulations. This would allow us to determine whether a robust range of spectral values exists, capable of characterizing universality classes in 3D weak turbulence effectively.

## Conclusion and future work

This paper proposes a new approach for studying 3D vortex dynamics. The approach, introduced as *gradient vortex dynamics* (GVD), is based on the visualization and characterization of $3D+1$ coherent structures using their respective gradient lattice. Our results, obtained from 3D weak turbulence dynamics, allow us to conclude that GVD presents the following analytical contributions: (i) it improves the visualization and monitoring of complex dynamics such as spirals defects, screws, irregular blobs and vortex rings; (ii) shows that the aspect ratio calculated directly on the gradient lattice is effective in characterizing quasi-stable regimes and their relaxation; (iii) the measurement of the phase-gradient allows robust characterization of nonlinear pattern oscillations in 3D; and (iv) the spectral analysis of the $G_{\phi } (t)$ time series brings a new perspective for characterizing complex regimes in the spatiotemporal domain with a lower mathematical-computational cost than other usual methods. Particularly the results achieved in this research show that the vortex dynamics in weak turbulence, via the Ginzburg-Landau model, involves four regimes: (i) regular blobs vorticity that evolves into (ii) inhomogeneous blobs inducing the formation of (iii) coherent structures with high helicity, such as screws and rings that tend to form (iv) larger mixed structures. The GVD technique allows us to identify in detail the formation of a screw during the third regime. The oscillations of phase turbulence have a characteristic frequency of 0.38 Hz, superimposed by high-frequency fluctuations whose spectral signature indicates a possible spatiotemporal chaotic behavior.

In subsequent research, we plan to investigate gradient vortex dynamics by simulating CGLE on larger grids, seeking to verify the phenomenological robustness of the results found in this work (classes of coherent structures, transients, metastability and regimes). We will also improve screw detection and analysis by incorporating a Lagrange approach to address the conjecture presented here. A stochastic component in the CGL model considering additive and multiplicative noises will be addressed. In addition, we will continue to explore and apply the most advanced technologies or theories, with emphasis on deep learning models, to enable automatic detection, classification, and diagnosis of coherent structures in complex three-dimensional systems. Applications in other systems, involving neutral fluids, MHD and chaotic coupled map lattices, are already underway, thus seeking to establish a new method, based on the 3D gradient, for characterizing three-dimensional nonlinear processes as turbulence and spatiotempoal chaos.

## Methods

### Numerical method stability for CGLE

To address the challenges of temporal instabilities inherent to the pseudo-spectral method, recent advancements have incorporated adaptive time-step strategies, improving both accuracy and computational efficiency in integration^74^. In this context, we have implemented the Runge-Kutta-Fehlberg 4-5 integration method^75^, which adjusts the integration time-step by comparing a 4th-order with a 5th-order parameter, according to an integration tolerance parameter $\tau$.

Our numerical code to CGLE can be accessed at

https://github.com/rsautter/3DCGLE/tree/main/CGL

### Gradient pattern analysis

Conceptual details on GPA can be found in precursor work on the technique^63,76–79^ well as its applications in the spatiotemporal context^19,39,63^ and, more recently, as a promising technique for image classification in astronomy^62,73,80^.

**Fig. 10:**
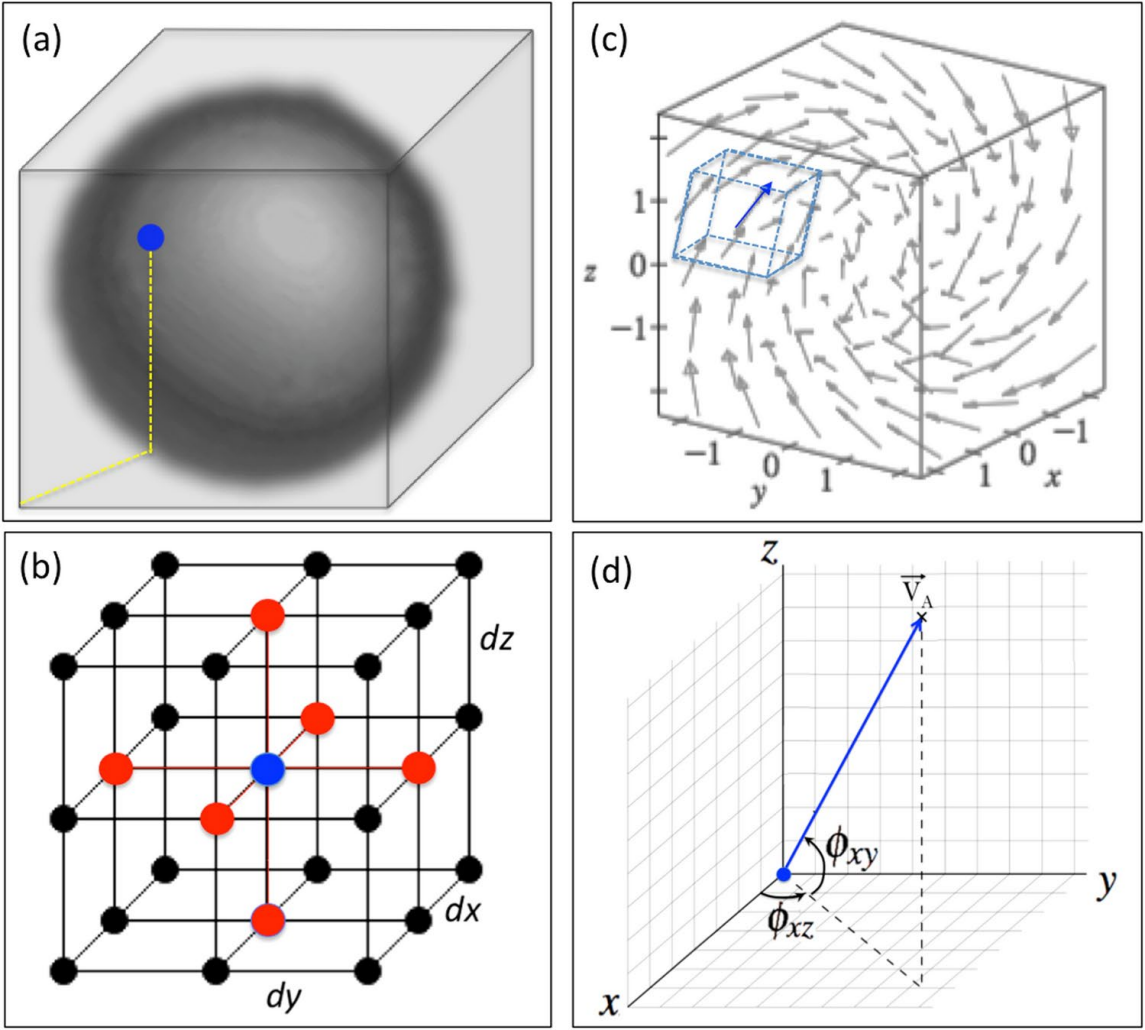
Gradient phase method: (**a**) A simple example of an amplitude hypercube where the operation to get the respective gradient hypercube will be performed. (**b**) The Central Finite Difference Grid performed on each amplitude in order to get the respective gradient lattice. (**c**) The respective gradient lattice from where the $G_{\phi }$, as defined in Eq. 2, is calculated. One of the vectors is highlighted (in blue) to exemplify how the phases (angles $\phi _{xy}$ and $\phi _{xz}$) are identified (**d**): Highlighting the gradient vector in the reference planes for measuring the phases to be input into Eq. 2.

For applications in 3D gradient phase dynamics, the first operation on an amplitude hypercube is to obtain its respective gradient hypercube. This is achieved through the centered finite difference technique that considers, in the 3D case, the six neighbors of the lattice (see Fig. 10 b). Once the gradients have been calculated (Fig. 10c), the angles $\phi _{xz}$ and $\phi _{xy}$ are obtained using spherical coordinates as shown in Fig. 10d. The method is the same used in GPA in 2D and its 3D version is detailed in Sautter 2023^50^. Note that the formula for $G_{\phi }$ (Equation 3) is derived directly from the third gradient moment adapted to the three-dimensional context represented in Fig. 10.

The code for applying GPA in 3D is available at: https://github.com/rsautter/3DCGLE/tree/main/GPA3D

### Computing $\Gamma _{\ell }$ from 3D snapshots

**Fig. 11:**
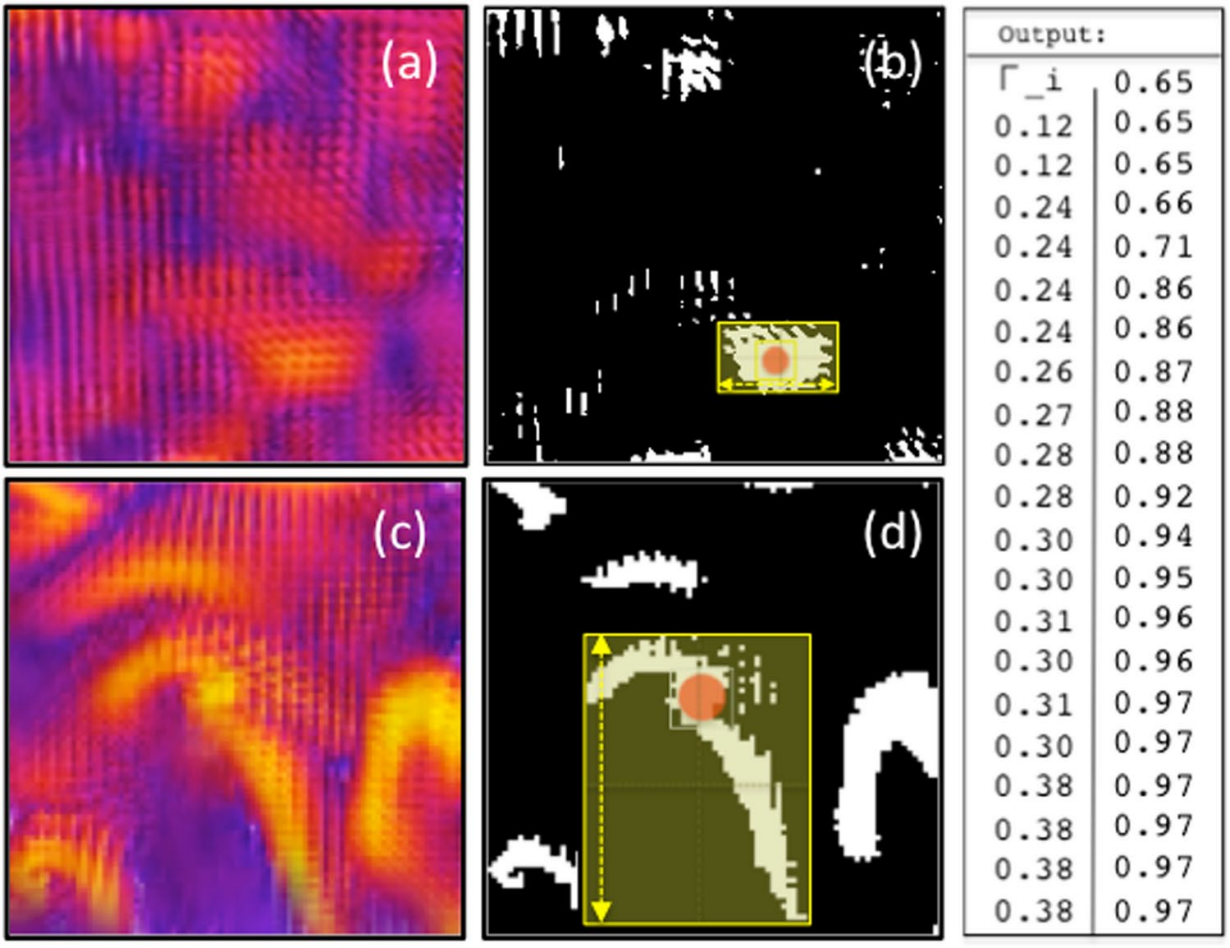
Examples of snapshots submitted to the $\Gamma _{\ell }$ obtaining method: (**a**) and (**c**) snapshots 130 and 730, respectively; (**b**) and (**d**) their respective binary counterparts with the identification of the selected substructure to obtain the linear scale $\ell$ for calculating the aspect ratio.

The aspect ratio at the lowest computational cost was defined as the largest linear size ($\ell$) of the substructure in relation to the size of the side of the hypercube (considered as the integral scale, *L*). The normalized ratio (taking $L=1$) is calculated in the binary image, treated in ENVI 6.8, and the calculation is done on the image in Python using ’dist’ from Scipy.spatial and CV2. The yellow squares are examples of coverage masks for the corresponding outputs (see Fig. 11). The aspect ratio can be obtained by the ratio between the areas or between the measurement of one of its sides in relation to the same measurement on the full scale (size of the binary image). Three examples are shown in Fig. 11, including all output values calculated for all representative snapshots that make up the curve shown in Fig. 6 (Up) that is the time series $\Gamma _{\ell }(t)$.

The steps to obtain the aspect ratio are as follows:

(i) Extract the projected 2D front image from the 3D hypercube at each snapshot time-step;
(ii) Find the region ($3\times 3$) of maximum brightness in the image;
(iii) Mark the center point of this region (the red ball in Fig. 8), as a mask ($3 \times 3$), on the binary image;
(iv) Adjust the mask by varying its vertical and horizontal sizes until the uniform contour boundary between 1 and 0 is found.
(v) Extract the greatest linear distance (yellow arrow in Fig. 8) from the mask (in centimeters);
(vi) Aspect ratio: divide this value by the linear size of the image (in centimeters).

The code for calculating the aspect ratio is implemented in IDL and can be made available by the authors upon request. The main libraries used, equivalent in Python, are available at: https://github.com/envi-idl/envipyengine

### Computing spectral indices

The spectral index is a measure that quantifies the scaling behavior of a signal in the frequency domain or in the autocorrelation scaling distribution. To calculate the spectral index of the time series extracted from the dynamics of the gradient hypercube, the PSD (Power Spectral Density) and Detrended Fluctuations Analysis (DFA) methods are considered.

The spectral index, $\beta$, via PSD is obtained as follows: (i) Compute Spectrum from the Fast Fourier Transform of the signal; (ii) Remove frequencies that are lower than 1/4 higher than 3/4 of the time series length. This allows a scaling balance between the lower frequencies, which often represent inertial motion or long-term trends, and the higher frequencies, which capture the fina structures fluctuations; (iii) Square the magnitude of each frequency component to obtain the power spectral density $P(k) \sim k^{-\beta }$ (alternatively, the wavenumber *k* is used instead of the frequency); (iv) A linear fit of the log-log plot between PSD and *k* is computed to determine the exponent $\beta$.

The spectral index, $\alpha$, via DFA is obtained as follows: (i) Divide the time series: For each scale *s*, divide the time series into non-overlapping segments of length *s*; (ii) Calculate fluctuation: For each segment, calculate the fluctuation by subtracting a linear trend fitted to the data within that segment; (iii) Average fluctuations: Average the fluctuations across all segments for the same scale *s*; (iv) DFA index: determine the relationship between the average fluctuation and the scale *s* on a log-log plot. The slope of the line on this plot represents the DFA index $\alpha$. DFA quantifies the self-similarity of a time series across different autocorrelation scales. Although DFA and PSD offer different perspectives, their scaling exponents are related by $\alpha = (\beta +1)/2$. However, discrepancies can arise in certain cases^65^, making it important to measure both indices. As shown in several works (e.g.^67^) DFA is more stable for short series (containing less than $2^{10}$) than PSD.

The code for calculating the DFA is available in Matlab at: https://www.maxlittle.net/software/

The version used in this research, which incorporates PSD and DFA, is implemented in Python and the notebook can be shared via *google colaboratory* upon request to the authors.

### Estimating the type of structure in each snapshot

The prevalence of a given type of structure, as reported in Table 2, was obtained from the following steps: (i) The 3D image is loaded into ENVI 6.8 as a raster (amplitude and gradient); (ii) a hybrid filter (Sobel and Directional Gradient) is applied to highlight edges in different sub-regions determined by the aspect ratio (horizontal, vertical and diagonal); (iii) The structures that repeat are segmented with thresholding and (iv) the FEAC tool (Feature Extraction Attribute Calculator of Envi Raster Statistics NV5) is applied to automatically count the different structures (objects) detected. We used an ENVIIDL script licensed by Harris Geospatial Solutions. A Python version is also available https://github.com/envi-idl/envipyengine.

## Supplementary Information

Below is the link to the electronic supplementary material.

Supplementary Information 1 (MP4 4175 KB)

Supplementary Information 2 (MP4 15 MB)

## Data Availability

The data contained in the plots within this paper and other findings of this study are available from the corresponding author on reasonable request. The movies (Mv01-Figure 4 and Mv02-Figure 5) are available on YouTube, respectively: https://www.youtube.com/watch?v=tc63qzBIM7Ihttps://www.youtube.com/watch?v=YGdTQabxMBs All numerical codes (simulation, analysis and visualization) are available from the corresponding author upon reasonable request, excepted the following in the github repository: https://github.com/rsautter/3DCGLE
