# Correction: Gradient vortex dynamics in 3D-weak turbulence

**DOI:** 10.1038/s41598-025-33407-7

**Published:** 2025-12-23

**Authors:** Rubens A. Sautter, Reinaldo R. Rosa, Debora C. Alavarce, Kristian T. Spoerer, Flavio H. Fenton

**Affiliations:** 1 Academic Department of Informatics, UTFPR, Pato Branco, PR 85503-390 Brazil; 2 Lab for Computing and Applied Mathematics, COPDT-INPE-MCTI, S.J. dos Campos, SP 12245-010 Brazil; 3 HIPOCAMPUS, São José dos Campos, 12246-900 Brazil; 4 School of Computer Science, University of Nottingham, Nottingham, 12245-010 UK; 5 School of Physics, Georgia Institute of Technology, Atlanta, 12245-010 USA

Correction to: *Scientific Reports* 10.1038/s41598-025-94832-2, published online 30 September 2025

In the original version of the Article, Figure 5 was a duplication of Figure 4. The legend was correct at the time of the publication.

The original Figure 5 and accompanying legend appear below.

**Fig. 5:**
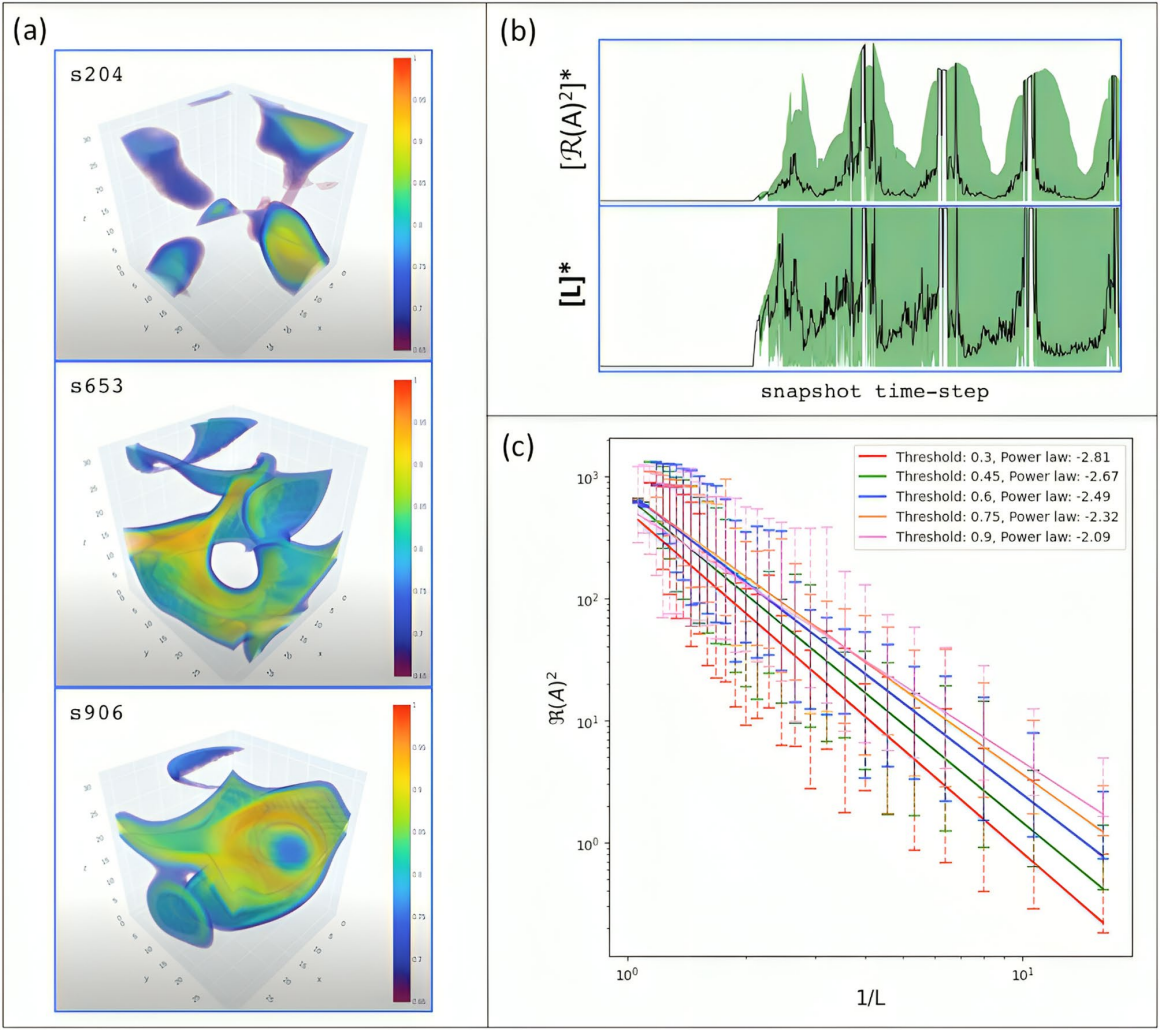
Gradient Hypercubes of (3*D* + 1)-CGLE weak turbulence obtained from our simulations. The complete time sequence is shown in *Movie Mv02* (available as a supplementary material). Each gradient hypercube represents the output of the CGLE simulation. The simulation renders 1000 snapshots with a total duration of 30s in real time. The snapshots were selected considering the identification of four main regimes for the variation of (Γℓ): (I) initial regime with the formation of quasi-regular blobs with Γ_ℓ_ < 0.25 for the range (0-175) (**a-c**); (II) secondary regime characterized by inhomogeneous blobs with low vorticity with 0.25 < Γ_ℓ_ < 0.33 for the range (176-325) (**d-f**); (III) high vorticity regime characterized mainly by screws and vortex rings with 0.33 < Γ_ℓ_ < 0.75 for the range (326-575) (**g-i**); (IV) regime with vortex rings and inhomogeneous blobs with Γ_ℓ_ close to one for the final range (576-1000) (**j-l**). To monitor all the dynamics and inspect morphological details in particular snapshots, we recommend interacting with the data in video format (see supplementary material).

The original Article has been corrected.

